# Transcriptional and metabolic responses of apple to different potassium environments

**DOI:** 10.3389/fpls.2023.1131708

**Published:** 2023-03-10

**Authors:** Tingting Sun, Junke Zhang, Qiang Zhang, Xingliang Li, Minji Li, Yuzhang Yang, Jia Zhou, Qinping Wei, Beibei Zhou

**Affiliations:** ^1^ Beijing Academy of Agriculture and Forestry Sciences, Beijing Academy of Forestry and Pomology Sciences, Beijing Engineering Research Center for Deciduous Fruit Trees, Key Laboratory of Biology and Genetic Improvement of Horticultural Crops, Ministry of Agriculture and Rural Affairs, Beijing, China; ^2^ College of Horticulture, China Agricultural University, Beijing, China

**Keywords:** apple, potassium deficiency, potassium excess, transcriptome analysis, metabolome analysis

## Abstract

Potassium (K) is one of the most important macronutrients for plant development and growth. The influence mechanism of different potassium stresses on the molecular regulation and metabolites of apple remains largely unknown. In this research, physiological, transcriptome, and metabolite analyses were compared under different K conditions in apple seedlings. The results showed that K deficiency and excess conditions influenced apple phenotypic characteristics, soil plant analytical development (SPAD) values, and photosynthesis. Hydrogen peroxide (H_2_O_2_) content, peroxidase (POD) activity, catalase (CAT) activity, abscisic acid (ABA) content, and indoleacetic acid (IAA) content were regulated by different K stresses. Transcriptome analysis indicated that there were 2,409 and 778 differentially expressed genes (DEGs) in apple leaves and roots under K deficiency conditions in addition to 1,393 and 1,205 DEGs in apple leaves and roots under potassium excess conditions, respectively. Kyoto Encyclopedia of Genes and Genomes (KEGG) pathway enrichment showed that the DEGs were involved in flavonoid biosynthesis, photosynthesis, and plant hormone signal transduction metabolite biosynthetic processes in response to different K conditions. There were 527 and 166 differential metabolites (DMAs) in leaves and roots under low-K stress as well as 228 and 150 DMAs in apple leaves and roots under high-K stress, respectively. Apple plants regulate carbon metabolism and the flavonoid pathway to respond to low-K and high-K stresses. This study provides a basis for understanding the metabolic processes underlying different K responses and provides a foundation to improve the utilization efficiency of K in apples.

## Introduction

Potassium (K) is one of the most essential macronutrients for plant growth and development, and it has essential physiological functions, such as plant osmoregulation, photosynthesis, protein synthesis, ion homeostasis, and enzyme activation (Kanai et al., 2011; Hafsi et al., 2014). Four forms of K exist in the soil, namely, exchangeable K, soluble K, lattice K, and fixed K. Only soluble K can be taken up by plants from the soil. The K concentration in the soil ranges from 0.1 to 6.0 mmol L^−1^ (Zeng et al., 2015). Too high or too low K concentrations in soil affect plant growth; in many regions, K concentrations are lower than 0.3 mmol L^−1^, and K deficiency limits plant growth (Schroeder et al., 1994). Under low-K conditions, the most common phenomena include stunted growth of plants, yellowing of leaf margins, and yield reduction (Hasanuzzaman et al., 2018). Excessive use of potassium fertilizer causes high-K stress, and excessive application of potassium fertilizer in soil causes soil and water pollution, reducing the productivity of crops. However, plants initiate a series of physiological processes as well as molecular and metabolite mechanisms to adapt to different levels of K stress. K deficiency and excess conditions are typical abiotic stress forms that induce a series of biological responses. Under different K stress conditions, reactive oxygen species (ROS) and phytohormones are affected (Ashley et al., 2006; Amtmann et al., 2008). Plant responses to different K conditions are also due to various complex gene regulatory networks that cause widespread changes in gene expression and metabolite contents (Liang et al., 2013).

Transcriptomes comprehensively and efficiently reveal gene expression, thereby allowing elucidation of the plant molecular mechanism response to different K stresses. In plants, many studies have focused on K uptake, loading, and transport mechanisms. Some related genes have been studied, such as the high-affinity K transporter/uptake transporter (HAK/KUP/KT) family, including AtHAK1/5, PpHAK2, AtHAK5, HvHAK1, OsHAK1, AtKUP3, AtKUP1, and OsHKT2, as well as shaker-like K channels (AKT), including OsAKT1 and AtAKT1/5 (Kim et al., 1998; Banuelos et al., 2002; Xu et al., 2006; Fulgenzi et al., 2008; Jung et al., 2009; Pyo et al., 2010; Kim et al., 2012; Oomen et al., 2012; Wu et al., 2019; Wang et al., 2021). These genes play a vital role in plants’ responses to different K conditions. Transcriptomic analysis of the response of *Arabidopsis*, rice, maize, soybean, sugarcane, and wild barley to K deficiency conditions has indicated that genes involved in metabolism, signal transduction, and ion transport are altered at the transcript level (Armengaud et al., 2004; Ma et al., 2012; Wang et al., 2012a; Zeng et al., 2014).

Metabolomics, known as qualitative and quantitative analysis of cellular metabolites, has become an important complementary tool for the study of plant functional genomics and systems biology (Weckwerth, 2003). Metabonomic analysis reflects the synthesis, decomposition, or transformation rules of some objects, all metabolites, or some metabolites in the tissue or cell (Hall, 2011). Abiotic stress causes changes in the expression of metabolic products, resulting in metabolite disorders *in vivo* (Meena et al., 2017). Many studies have reported the changes in small-molecule compounds in response to mineral nutritional stress. Sung et al. (2015) reported metabolic responses to deficiencies in nitrogen (N), phosphorus (P), and K, and they demonstrated that the lack of these elements decreases energy production and amino acid metabolism in tomato leaves and roots. Low-K stress increases monosaccharide, disaccharide, polysaccharide, and putrescine contents in barley (Zhao et al., 2021). The contents of putrescine, aconitate, citrate, malate, and fumarate increased in sunflower under low-K stress (Cui et al., 2019). Citric acid, arginine, and asparagine contents are upregulated under K deficiency in rapeseed leaves (Hu et al., 2021). The levels of glutamic acid and aspartic acid are decreased in peanut under low-K conditions, whereas the levels of histidine, lysine, and arginine are increased in peanut under low-K conditions (Patel et al., 2022). The amino acid contents are increased in both K-sensitive and K-tolerant genotypes of wheat roots under K starvation (Zhao et al., 2020).

Apple (*Malus domestica*) is one of the most important fruits in the world, and apple production and consumption are the highest in China. K fertilizer plays a key role in apple growth and ripening. When K deficiency occurs in apple trees, the middle and lower leaves of new shoots turn yellow. In severe cases, the leaves gradually show brown withered spots, resulting in a curly scorched appearance, and new shoots stop growing early, forming small flower buds and small fruits with a color difference and a decline in quality (Chang et al., 2014). High-K stress causes the occurrence of apple bitter pox, which reduces the absorption of cations, such as calcium and magnesium, by plants, thus affecting the yield of plants. Excessive application of potassium fertilizer causes soil environmental pollution and water pollution. Many studies have investigated the molecular mechanisms that occur under K deficiency in model plants, such as *Arabidopsis*, rice, and maize (Armengaud et al., 2004; Ma et al., 2012; Wang et al., 2012a). However, these mechanisms have rarely been reported in apple, especially under high-potassium stress. In the present study, we investigated the molecular response mechanism and metabolite changes of apple to low-potassium and high-potassium stresses, and we provided a theoretical basis for further study on the response mechanism of apple to potassium.

## Materials and methods

### Plant growth conditions

The experimental materials, namely, “CG-935” apple seedlings, were tissue cultured, after rooting, seeding, and transplantation, and the apple plants were then transported to the experimental field as previously reported by Sun et al. (2021).

### Different potassium treatments

After 90 days, healthy apple seedlings of similar size (with 16–20 leaves) were transferred to a hydroponic slot (60 × 37 × 35 cm) containing 60 L of a 1/2-strength Hoagland nutrient solution (Hoagland and Arnon, 1950). Stress treatments were initiated after 10 days of precultivation. Apple seedlings were randomly divided into 3 groups with 54 plants per treatment, and there were three biological replicates for each stress treatment with 18 plants per replicate. The treatments were as follows: (1) control (CK), 1/2-strength Hoagland nutrient solution supplemented with 3 mM K_2_SO_4_; (2) low-K treatment (LK), 1/2-strength Hoagland nutrient solution with 50 μM K_2_SO_4_; and (3) high-K treatment (HK), 1/2-strength Hoagland nutrient solution with 15 mM K_2_SO_4_ (Chang et al., 2014). The solution was continuously aerated and refreshed every 3 days, and the experimental treatment lasted for 15 days. Plant roots and leaves were harvested for physiological, transcriptomic, and metabolomic analyses. The samples were designated as follows: the apple leaves and roots in the control condition were named CKL and CKR, respectively; the apple leaves and roots in the low-K condition were named LKL and LKR, respectively; and the apple leaves and roots in the high-K condition were named HKL and HKR, respectively.

### Growth indices, photosynthetic indices, and nutrient concentration measurements

After 15 days of treatment, the plant heights, stem diameters, and dry weights (DWs) of the whole apple as well as the ratio of underground DW to aboveground DW (R/S) of the apple were calculated.

The net photosynthetic rate (Pn), transpiration rate (Tr), water use efficiency indicator (WUEi), stomatal conductance (Gs), and intercellular CO_2_ concentration (Ci) values of the apple leaves were measured by an LI-6800 portable photosynthesis system (LI-COR Inc., Lincoln, NE, USA) on sunny days.

The measurements of soil plant analytical development (SPAD) of apple leaves and the concentrations of N, P, and K in apple roots, apple stems, apple leaves, and whole apple were measured according to the method of Sun et al. (2021).

### Determination of H_2_O_2_, enzyme activities, and phytohormones

For the determination of H_2_O_2_, enzyme activity, and phytohormones, nine apple seedlings were selected for each experimental replicate (*n* = 3) to provide an adequate amount of root and leaf tissue. The levels of hydrogen peroxide (H_2_O_2_, SO1300), and the activities of peroxidase (POD, KT5058), catalase (CAT, KT4957), abscisic acid (ABA, KT4924), and indoleacetic acid (IAA, NR, KT4992) were determined using commercial test kits purchased from Jiangsu Kete Biotechnology Co., Ltd. (Jiangsu, China). H_2_O_2_ was recorded on a UV-1750 spectrometer (Shimadzu, Japan). The enzyme activities were analyzed using an ELISA reader (Multiskan MS, Labsystems 325, Helsinki, Finland).

### RNA isolation, qRT-PCR analysis, and transcriptome sequencing

Total RNA of apple leaves and roots was isolated using TRIzol Reagent (Invitrogen, Carlsbad, CA, USA) for quantitative real-time PCR (qRT-PCR) analysis and RNA sequencing (RNA-seq) analysis, and three biological replicates of each sample were sequenced. Quantitative real-time PCR (qRT-PCR) analysis was conducted according to Sun et al. (2021), and the primers used for qRT-PCR are listed in **Table S1**. Transcriptome analysis was performed by Wuhan MetWare Biotechnology Co., Ltd. (www.metware.cn, Wuhan, China). After rapid filtering (version 0.18.0) (Chen et al., 2018), the HISAT2.2.4 and Bowtie2 tools were used to compare clean reads with the apple genome (https://iris.angers.inra.fr/gddh13/index.html) (Langmead et al., 2009; Kim et al., 2015). The RESM software was used to calculate the values of fragment per kilobase of transcript per million mapped reads (FPKM) (Li and Dewey, 2011). Differentially expressed genes (DEGs) were determined according to cutoffs of log2(fold change) ≥ 1 and *p* ≤ 0.05. Kyoto Encyclopedia of Genes and Genomes (KEGG) and Gene Ontology (GO) tools were used to analyze the DEGs.

### Metabolite analysis

Metabolites were extracted and analyzed at Wuhan MetWare Biotechnology Co., Ltd., Wuhan, China (www.metware.cn) (Zhang et al., 2019). The metabolite analysis was performed using a liquid chromatography–electrospray ionization–tandem mass spectrometry (LC-ESI-MS/MS) system (HPLC, Shim-pack UFLC SHIMADZU CBM30A system; MS, Applied Biosystems 6500 Q TRAP). Metabolite quantification was performed using multiple reaction monitoring (MRM) in triple quadrupole mass spectrometry (Chen et al., 2013). Metabolomic data analysis was performed according to previous methods (Zhu et al., 2018).

### Statistical analysis

The statistical analysis of different plant treatments in triplicate (*n* = 3) was performed by one-way analysis of variance (ANOVA) using SPSS 20.0 software. A probability value of *p* < 0.05 was considered statistically significant. The data are presented as the mean ± standard deviation (SD) of three replicates.

## Results

### Potassium affects plant growth and mineral nutrients

After 15 days of treatment with 50 μM (LK treatment), 3 mM (CK treatment), or 15 mM (HK treatment) K_2_SO_4_ in hydroponic culture, the plant height, stem diameter, DW, and root/shoot ratio decreased under K stress. The plant height decreased more in LK (82.49%) than in HK (82.73%), and the stem diameter also showed a similar trend. The DW of LK-treated apple plants was 72.94% of that of CK plants, and the DW of HK-treated seedlings was 75.69% of that of CK seedlings. The root/shoot ratio significantly increased by 111.76% in LK but decreased by 65.5% in HK (**Table 1**).

**Table 1:** The growth indexes in apple under control (CK), low-potassium (LK), and high-potassium (HK) conditions.

Treatment	Plant height (cm)	Stem diameter (mm)	Dry weight (g/plant)	Root/Shoot
CK	18.67 ± 0.89 a	3.92 ± 0.23 a	2.18 ± 0.31 a (100)	0.34 ± 0.02 b
LK	15.40 ± 1.10 b	3.31 ± 0.16 b	1.59 ± 0.21 b (72.94)	0.38 ± 0.01 a
HK	17.50 ± 0.68 a	3.72 ± 0.15 a	1.65 ± 0.19 b (75.69)	0.28 ± 0.03 c

Data indicate means ± SE (*n* = 3). Different letters beside the values in the same column indicate significant difference between the treatments.

The value of SPDA is a reliable indicator that can represent the content of chlorophyll (Tan et al., 2021). The SPDA values and photosynthetic characteristics were different under LK and HK stresses. The SPAD significantly decreased under LK, but there was no difference under HK. The Pn, Tr, WUEi, and Gs were significantly reduced under LK and HK, especially under K deficiency conditions (**Table 2**).

**Table 2:** The photosynthetic characteristics in apple under control (CK), low-potassium (LK), and high-potassium (HK) conditions.

Treatment	SPAD	Pn (µmol CO_2_/m^2^/s)	Tr (mmol H_2_O/m^2^/s)	WUEi (µmol/mmol)		Gs (mol H_2_O/m^2^/s)	Ci (µmol CO^2^/mol)
CK	49.07 ± 2.74 a	12.73 ± 0.59 a	2.80 ± 0.09 a	4.55 ± 0.22 a		0.36 ± 0.01 a	282.88 ± 8.83 a
LK	43.98 ± 2.12 b	8.00 ± 0.56 c	2.24 ± 0.12 c	2.59 ± 0.25 b		0.17 ± 0.02 c	281.48 ± 12.02 a
HK	49.59 ± 2.76 a	9.75 ± 0.84 b	2.61 ± 0.07 b	2.26 ± 0.23 b		0.29 ± 0.01 b	285.44 ± 14.18 a

Data indicate means ± SE (*n* = 3). Different letters beside the values in the same column indicate significant difference between the treatments.

Differences in K fertilization conditions were reflected in the apple root, stem, and leaf elemental N, P, and K mineral nutrients. The concentrations of K in apple trees were lower under LK stress but higher when more K was available, and the P and K concentrations were much higher in CK compared to HK and LK (**Table S2**).

### H_2_O_2_ content, enzyme activities, and phytohormones

**Figure 1:**
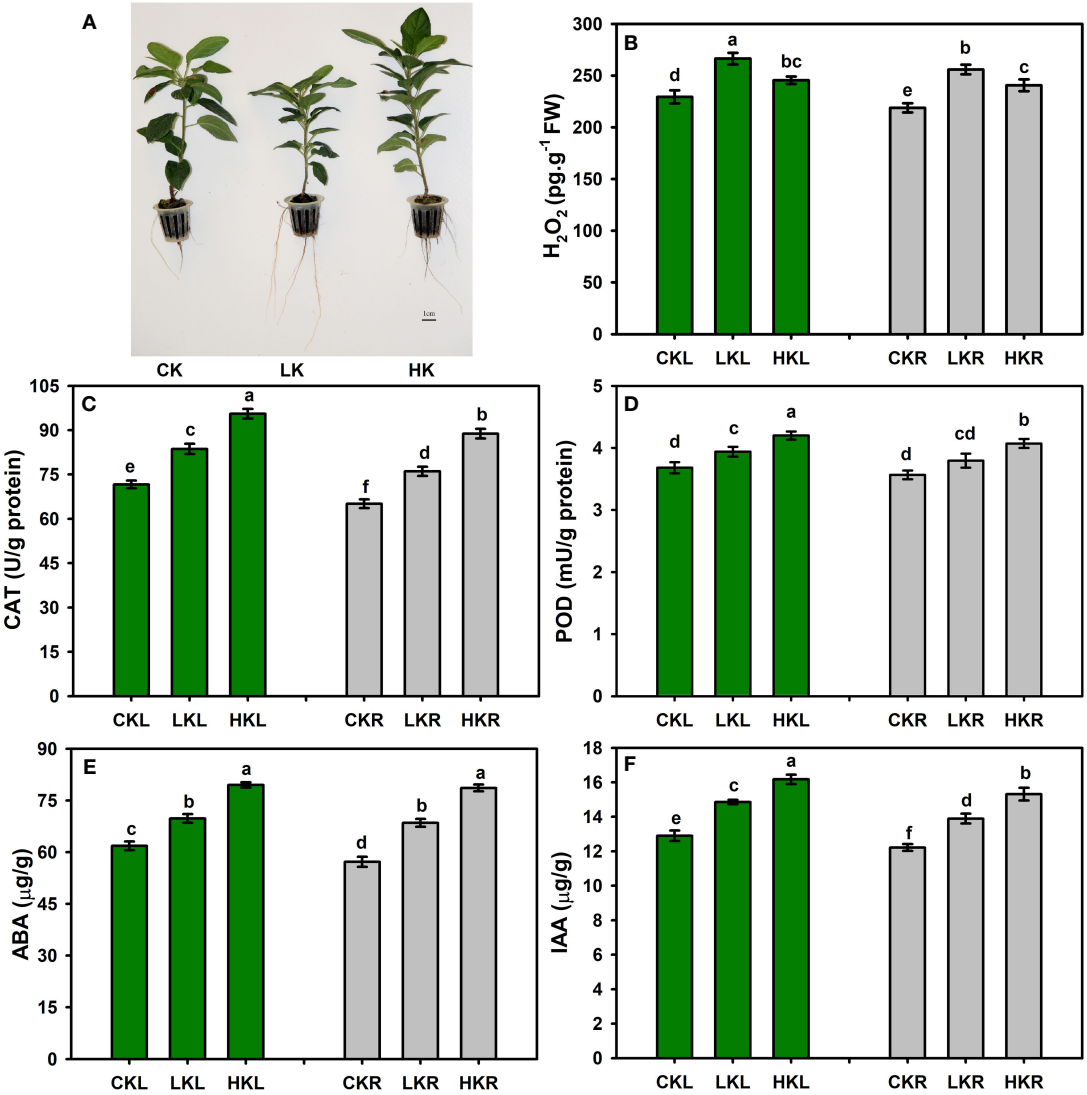
Measurement of H_2_O_2_, enzyme activities, and phytohormones in apple under different K treatments. **(A)** The phenotypic characteristics of apple. **(B)** The content of H_2_O_2_. The activities of CAT **(C)** and POD **(D)**. The contents of ABA **(E)** and IAA **(F)**. Different letters indicate significant differences according to Tukey’s multiple-range tests (p < 0.05).

Plant biomass decreased under K stress, while root growth increased under LK stress (**Figure 1A**). The H_2_O_2_ content, superoxide dismutase (SOD) activity, and POD activity were affected by different K stresses. The H_2_O_2_ content increased in apple leaves and roots under the LK and HK treatments with greater increases under LK stress. Under different K conditions, the enzyme activities increased in both apple leaves and roots, but the range of increase varied. The activities of SOD and POD significantly increased under different K conditions. The ABA content increased by 1.13- and 1.29-fold in apple leaves under LK, and it increased by 1.14- and 1.25-fold in roots under HK (**Figure 1E**). The IAA content exhibited a similar trend (**Figure 1F**).

### Differential gene expression analysis

To obtain a global overview of the transcriptome responses to different K treatments in apple roots and leaves, four RNA-seq libraries were prepared, namely, HKL/CKL, HKL/CKL, LKR/CKR, and HKR/CKR, according to different K stress conditions in apple roots and leaves. The transcriptome data of the 18 samples described in the study have been deposited into the National Center for Biotechnology Information (NCBI) databases, and the bioproject accession number is PRJNA895870. The sequencing data are summarized in **Table S3**. The average for each sample of clean reads was approximately 4.5 × 10^7^, and the sequence alignment efficiency ranged from 82.59% to 90.04%. The FPKM of HK was higher than those of CK and LK, and FPKM > 1 was used as the threshold to determine gene expression (**Figure S1A**). The Pearson correlations among the LK, HK, and CK replicates ranged from 0.97 to 1 in leaves and from 0.98 to l in roots (**Figure S1B**). Principal component analysis (PCA) indicated that the roots and leaves of LK, HK, and CK were clustered together, indicating significant differences in gene expression profiles. The LK, HK, and CK in leaf and root replicates were not tightly clustered, showing that inoculation occurred within the replicates (**Figure S1C**).

According to DESeq2 analysis using cutoffs of |log 2-fold change| ≥ 1 and false discovery rate (FDR) < 0.05, a total of 2,409 transcripts were differentially expressed in apple leaves under LK with 1,412 upregulated genes and 997 downregulated genes (**Figure S2A**). A total of 1,393 DEGs were detected under HK conditions with 829 upregulated DEGs and 564 downregulated DEGs in apple leaves under HK (**Figure S2B**). These observations suggested that under different K conditions, more DEGs were found under LK conditions than under HK conditions in apple leaves. A total of 778 DEGs were differentially expressed in apple roots under LK stress with 442 upregulated genes and 336 downregulated genes (**Figure S2C**), and a total of 1,205 DEGs were differentially expressed in apple roots under HK with 819 upregulated DEGs and 386 downregulated DEGs (**Figure S2D**). In terms of fold change gene expression values, the following maximum upregulation and maximum downregulation values were observed: 8.19 log_2_ FC and −8.38 log_2_ FC in leaves under LK, respectively; 7.92 and −7.42 log_2_ FC in leaves under HK, respectively; 7.59 and −13.15 log_2_ FC in roots under LK, respectively; and 8.24 and −4.64 log_2_ FC in roots under HK stresses, respectively (**Figure S2E**).

**Figure 2:**
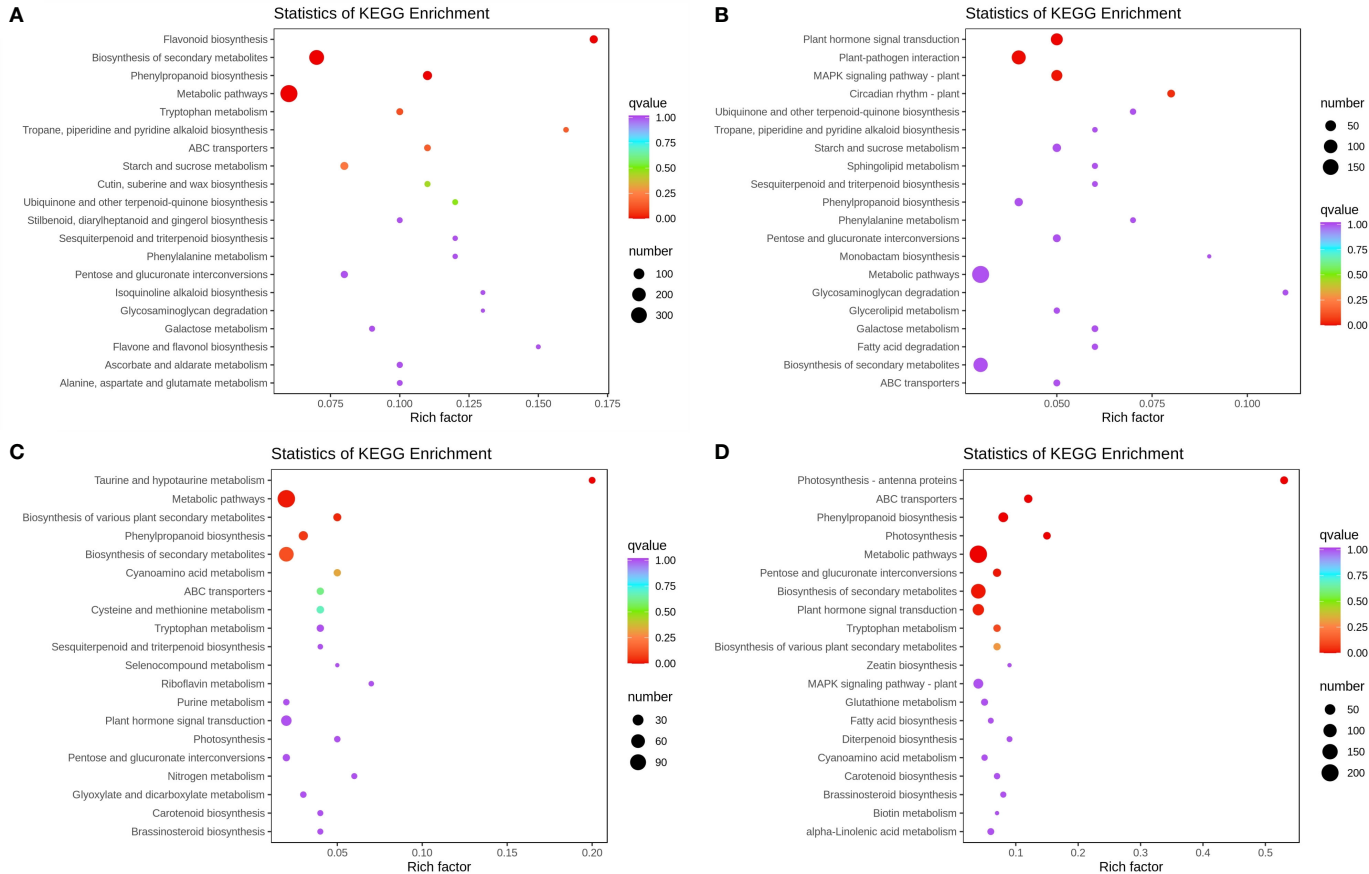
Statistics of KEGG enrichment under different K conditions in apple leaves and roots. **(A)** Statistics of KEGG enrichment under low-K conditions in apple leaves. **(B)** Statistics of KEGG enrichment under high K conditions in apple leaves. **(C)** Statistics of KEGG enrichment under low-K conditions in apple roots. **(D)** Statistics of KEGG enrichment under high K conditions in apple roots.

GO annotation analysis showed enrichment classifications according to biological processes (BPs), molecular functions (MFs), and cellular components (CCs) (**Figure S3**). **Figure 2** shows the top 20 significantly enriched pathways under different K stresses in apple leaves and roots. KEGG pathway enrichment revealed that the vital biological pathways in response to different K conditions were involved in flavonoid biosynthesis, photosynthesis, plant hormone signal transduction, and biosynthesis of various plant secondary metabolite biosynthetic processes.

The DEGs involved in potassium metabolism were then thoroughly analyzed. The DEGs associated with potassium uptake, loading, and transport processes were detected in four pairs of libraries (**Table 3**). Most of the potassium transporter gene family members were increased in the apple seedlings.

**Table 3:** Genes encoding transporters showed differential expression in response to different K stresses.

Gene	Seq ID	LKL	HKL	LKR	HKR
AKT	MD15G1178200			1.13	
KAT	MD05G1284400	1.18			
KUP	MD16G1089900	−1.18			
	MD01G1165900		1.04		
	MD07G1232700		1.04		
HAK	MD03G1283600				2.89
	MD10G1204500				3.37
	MD11G1302600			1.71	1.18
	MD11G1303100			1.91	1.40
	MD11G1302900			2.29	
	MD13G1133200			1.32	
	MD16G1143900			1.34	

### qRT-PCR validation of the DEGs

Ten apple genes were detected by qRT-PCR for expression analysis to validate the RNA-seq results. The RT-qPCR analysis results were not significantly different from the RNA-Seq data, and similar trends were found in the up- and downregulated genes (**Figure S4**).

### Metabolomic response to different K treatments in apple

**Figure 3:**
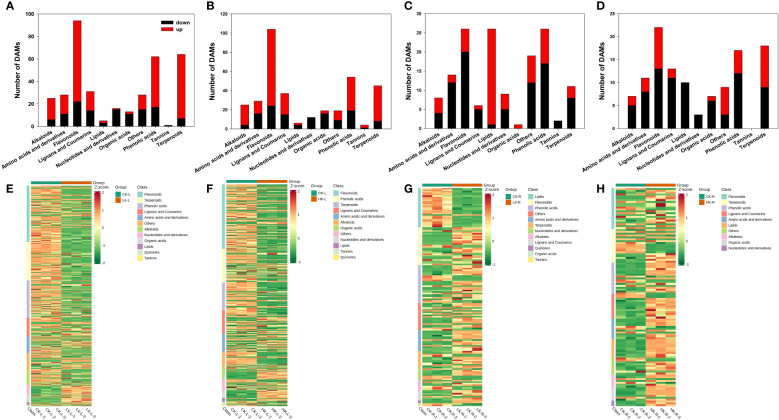
Differentially accumulated metabolites among different potassium treatments in apple. **(A, E)** Major classes of detected metabolites and heatmaps of differential metabolites between LKL/CKL. **(B, F)** Major classes of detected metabolites and heatmaps of differential metabolites between HKL/CKL. **(C, G)** Major classes of detected metabolites and heatmaps of differential metabolites between LKR/CKR. **(D, H)** Major classes of detected metabolites and heatmaps of differential metabolites between HKR/CKR. Three independent replicates of each stage are also displayed in the heatmap.

The metabolome of apple seedlings was analyzed using four different pairs of libraries, namely, LKL/CKL, HKL/CKL, LKR/CKR, and HKR/CKR. Overall, 527 DAMs were quantified and identified in LKL/CKL with 246 upregulated DAMs and 122 downregulated DAMs. For HKL/CKL, 228 DAMs were significantly upregulated, and 129 DAMs were downregulated. For LKR/CKR, 166 DAMs were identified with 74 upregulated DAMs and 92 downregulated DAMs. For HKR/CKR, 150 DAMs were identified with 92 upregulated DAMs and 74 downregulated DAMs (**Table S4**). The heatmaps of the differences in metabolites among the four combinations show the above trends (**Figure 3**).

Under different K stresses, the contents of amino acids, amino acid derivatives, organic acids, carbohydrates, flavonoids, and lipids changed. Under LK conditions, most lipids, flavonoids, and phenolic acids increased in apple roots. The metabolites of apple leaves and roots under different potassium stress conditions are presented in **Figure 3**. In apple leaves, the content of amino acids, amino acid derivatives, phenolic acids, terpenoids, and flavonoids increased under different K stresses, while nucleotides and nucleotide derivatives decreased. Under LK conditions, the content of lipids was upregulated in apple roots.

**Figure 4:**
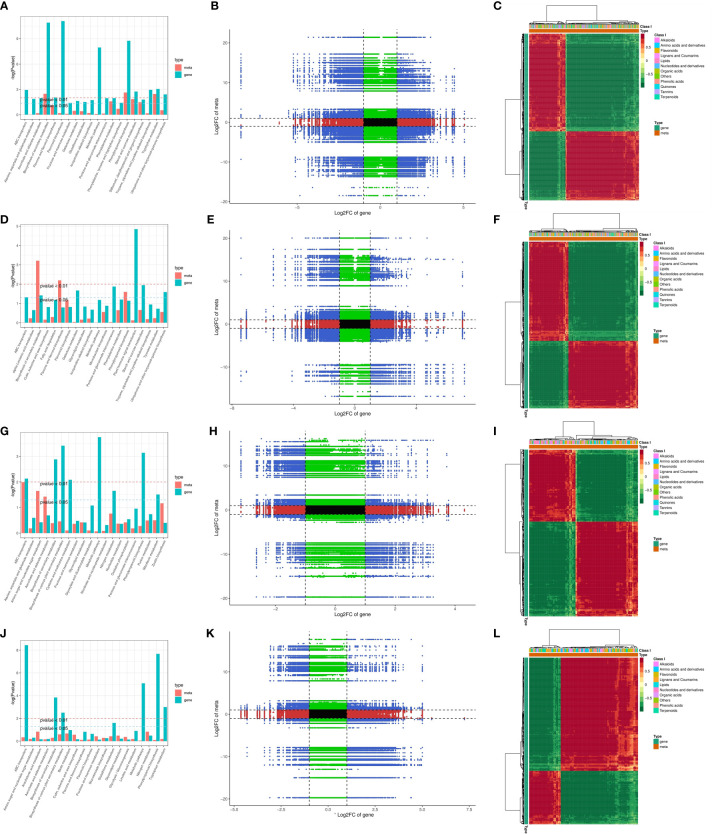
**(A, D, G, J)** Histograms of joint KEGG enrichment *p*-values, and **(B, E, H, K)** the associations of transcriptomic and metabolomic variation quadrant diagrams in LKL/CKL, HKL/CKL, LKR/CKR, and HKR/CKR; the black dotted lines indicate the differential thresholds. Outside the threshold lines, there were significant differences in the gene/metabolites, and within the threshold lines are shown the unchanged gene/metabolites. Each point represents a gene/metabolite. Black dots, green dots, red dots, and blue dots indicate unchanged genes/metabolites, differentially accumulated metabolites with unchanged genes, differentially expressed genes with unchanged metabolites, and both differentially expressed genes and differentially accumulated metabolites, respectively. **(C, F, I, L)** Heatmaps of the correlation coefficient clusters (>0.8), *p*-values < 0.05.

The co-joint KEGG enrichment analysis determined the co-mapped pathways in apple leaves and roots under K deficiency and excess conditions (**Figures 4A, D, G, J**). Of the metabolic pathways, the co-mapped pathways, namely, flavonoid biosynthesis, carbon metabolism, biosynthesis of secondary metabolites, glycerolipid biosynthesis, and phenylpropanoid biosynthesis, were the significantly enriched pathways under different K stresses. The Pearson correlation coefficients for the nine quadrants are shown in **Figures 4B, E, H, K**. In the third and seventh quadrants, the gene and metabolite differential expression patterns were consistent; the genes were positively correlated with the regulation of metabolites, and the changes in metabolites were positively regulated by the genes. The DEGs and DAMs with Pearson correlation coefficients (PCCs) higher than 0.8 were further selected and represented by heatmaps (**Figures 4C, F, I, L**).

### Responses of carbon metabolite and flavonoid metabolites in apple to different potassium conditions

**Figure 5:**
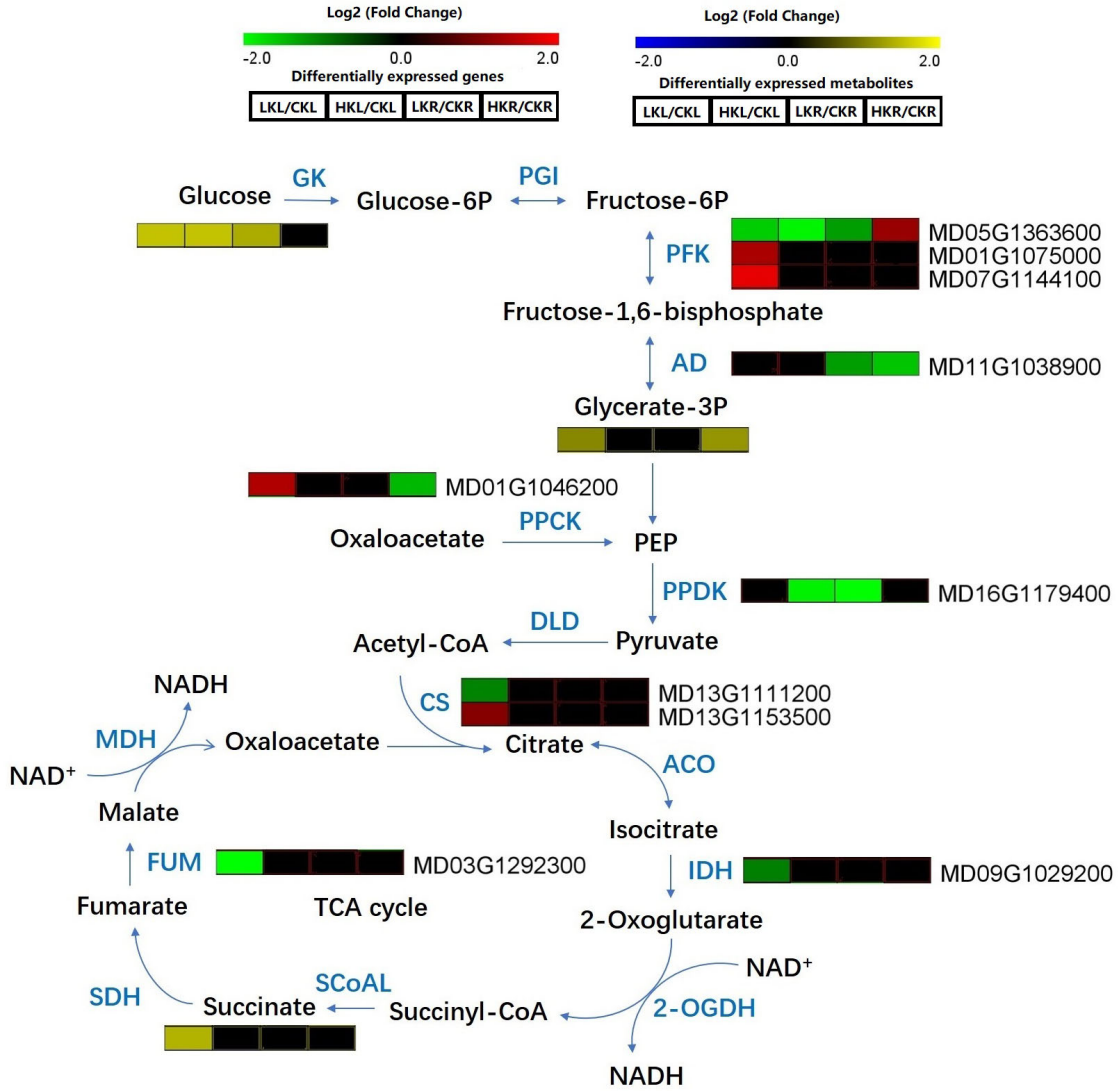
Carbon metabolites in apple leaves and roots under different K stresses. The boxes in the pathway represent DEGs or DAMs. Red and green represent upregulated and downregulated genes, respectively. Yellow and blue represent upregulated and downregulated metabolites, respectively.

The concentrations of glucose, glycerate-3P, and succinate, which are involved in carbon metabolism, particularly glycolysis and the tricarboxylic acid (TCA) cycle, were increased in apple leaves under LK. The glucose content was upregulated in LKR/CKR and HKL/CKL, whereas glycerate-3P was increased in HKR/CKR (**Figure 5**). Under LK stress, the following changes were observed: the expression of *PFK* (MD05G13633600), *CS* (MD13G1111200), *FUM* (MD03G1292300), and *IDH* (MD09G1029200) was decreased in leaves; the expression of *PFK* (MD01G107500 and MD07G1144100) and *CS* (MD13G1153500) was increased in leaves; and the expression of *PFK* (MD 05G1363600) and *PPDK* (MD16G1179400) was decreased in roots. Under HP conditions, the expression of *PFK* (MD 05G1363600) and *PPDK* (MD16G1179400) was downregulated in leaves; the expression of *PFK* (MD05G1363600) was increased in roots; and the expression of *AD* (MD11G1038900) and *PPCK* (MD01G1046200) was decreased in roots (**Figure 5**).

**Figure 6:**
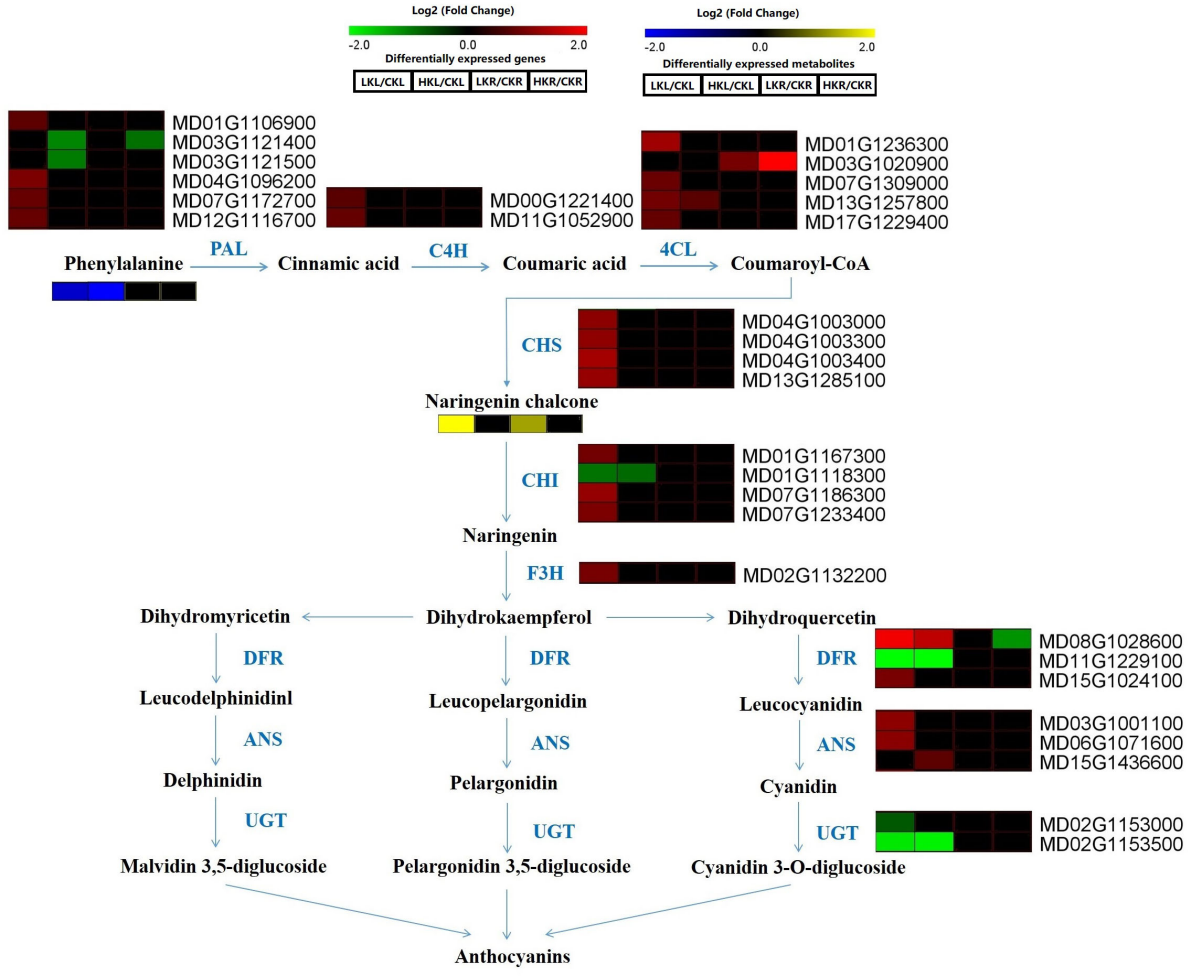
Flavonoid metabolites in apple leaves and roots under different K stresses. The boxes in the pathway represent DEGs or DAMs. Red and green represent upregulated and downregulated genes, respectively. Yellow and blue represent upregulated and downregulated metabolites, respectively.

The level of naringenin chalcone, which is involved in flavonoid metabolites, was increased under LK conditions in apple leaves and roots. The phenylalanine content was decreased in apple leaves under different K conditions. The gene expression of *PAL* in leaves was increased under LK stress but decreased under HK conditions. The *C4H*, *4CL*, *CHS*, *F3H*, *ANS*, *CHI*, and *DFR* genes were upregulated under LK stress, whereas the *UGT* gene was decreased under LK stress. Under HK conditions, the *PAL*, *UGT*, *CHI* (MD01G1118300), and *DFR* (MD11G1229100) genes were downregulated in leaves (**Figure 6**).

## Discussion

K plays essential roles in many physiological and biochemical processes in the plants, such as ion homeostasis, enzyme activation, osmoregulation, and protein synthesis (Kanai et al., 2007; Ma et al., 2020). K stress affects the normal growth of plants; the scarcity of K slows plant growth, reduces plant height, reduces stem diameter (Tester and Blatt, 1989), and decreases photosynthesis (Kanai et al., 2011). The plant height, stem diameter, plant DW, and photosynthesis were decreased under different LK and HK stresses in apple (**Tables 1**, **2**). Trankner et al. (2018) revealed that K deficiency also reduces photosynthetic CO_2_ fixation, as well as the transportation and consumption of photoassimilates, thus damaging plant membranes and chlorophyll under low-K conditions. In the present research, the SPDA value and photosynthetic characteristics decreased under LK stress in apple (**Table 2**). Xu et al. (2020) reported that an adequate supply of K increased the photoassimilate transportation rate from apple leaves to roots as well as increased nutrient use efficiency by influencing photosynthesis. The SPAD value of apple plants was slightly increased under HK stress, while the photosynthetic index decreased to a lesser extent under HK stress compared to LK stress (**Table 2**); it means that apple seedlings under low-potassium stress are more damaged than those under high-potassium stress. In the present study, RNA-Seq and KEGG enrichment analysis indicated that a different potassium environment had an effect on plant photosynthesis. Combined transcriptome metabolome analysis showed that the DEGs and DAMs were associated with biological processes, such as carbohydrate metabolism and photosynthesis. Hafsi et al. (2014) revealed that K stress limits plant leaf growth, which may be due to sugar starvation in stems and leaves. In the present study, genes involved in the TCA cycle, such as *CS*, *IDH*, and *TCA*, were downregulated in apple leaves under LK (**Figure 5**), which may induce apple plant growth restriction.

Plants under K stress conditions increase ROS production, resulting in oxidative stress (Hernandez et al., 2012). The accumulation of higher K in plant cells restores oxidative stress by increasing the activity of antioxidant enzymes, such as glutathione reductase (GPX), dehydroascorbate reductase (DHAR), ascorbate peroxidase (APX), CAT, SOD, and POD (García-Martí et al., 2019). The H_2_O_2_ content, SOD activity, and POD activity were affected by different K stresses in apple seedlings. The SOD and POD activities were significantly increased to combat different K conditions (**Figures 1B–D**). Under HK stress, the enzyme activities increased more significantly than under LK stress, which indicated that LK had a greater effect on apple plants. A previously metabolome analysis has revealed that the glutathione content is increased in roots in low-K-tolerant KN9204 wheat but not in low-K-sensitive BN207 wheat (Zhao et al., 2020). Under low potassium, the content of S-(methyl)glutathione in apple leaves and roots was significantly increased in the present study (**Table S4**). Thus, these findings indicated that glutathione is an important metabolite for plant adaptation to K deficiency.

Phytohormones are active substances that widely exist in plants to regulate their physiological metabolism, affect plant development, affect plant growth, and play a regulatory role in stress conditions. Different potassium environments influence phytohormones in plants, such as brassinosteroids, IAA, ABA, and jasmonic acid (JA) (Ahanger et al., 2018; Ahanger et al., 2020; Yang et al., 2020). After 15 days of different K stress treatments, ABA and IAA contents increased in both apple leaves and roots (**Figures 1E, F**). The concentration of ABA in peanut leaves also increases under low-K stress (Patel et al., 2022). Therefore, considering the importance of phytohormones in plant growth, these findings indicated that ABA, JA, SA, and other phytohormones are important molecules in plant resistance to K stress.

Potassium uptake and absorption are mainly accomplished through potassium transporters and potassium channels in the plasma membrane (Ashley et al., 2006). K transporters and channels play vital roles in translocation and cell growth in various plants (Wang and Wu, 2013). Under the condition of K deficiency, the expression levels of *HAK1* and *HAK5* in maize are upregulated, and *AtHAK5*, *OsHAK1*, and *HvHAK1* are also induced by K-limited conditions (Santa-María et al., 1997; Banuelos et al., 2002; Ahn et al., 2004; Gierth et al., 2005; Fulgenzi et al., 2008; Qin et al., 2019). In addition, transcriptome analysis of rice roots under LK stress has revealed that the *OsHAK1*, *OsHAK7*, *OsHAK11*, and *OsHKT2;1* genes are upregulated; in addition, potassium channel genes, such as *OsAKT1*, *OsAKT2/3*, and *OsKCO1*, are also increased in response to low-K conditions (Ma et al., 2012). In tobacco seedlings, the *KUP3* K transporter and the *SKOR* K channel are increased under LK stress (Lu et al., 2015). In apple seedlings, the *AKT* (MD15G1178200) and *HAK* (MD11G1302600, MD11G1303100, MD11G1302900, MD13G1133200, and MD16G1143900) genes were upregulated in roots under KL. Moreover, the *KAT* (MD05G1284400) and *KUP* (MD01G1165900 and MD07G1232700) genes were upregulated in apple leaves, whereas *HAK* (MD03G1283600, MD10G1204500, MD11G1302600, and MD11G1303100) was increased in apple roots (**Table 3**). These results were consistent with previous studies, showing that a common regulatory mechanism exists across plant species whereby the transcription of genes encoding K transporters and channels increases, which may be an efficient strategy to increase potassium uptake in plants under a K-deficient environment. Therefore, these findings suggested that apple *AKT*, *HAK*, *KUP*, and *HAK* are key genes involved in potassium channels and transporters, which play an important role in coping with low- and high-potassium stresses in apple seedlings.

Under potassium-deficient conditions, the amino acid content increases in cotton (Wang et al., 2012b) and in roots of the K-tolerant genotype of wheat (Zhao et al., 2020), and lysine, histidine, and arginine accumulated in peanut leaves and roots (Patel et al., 2022). In addition, the citric acid, arginine, and asparagine contents increase under LK in rapeseed (Hu et al., 2021), and most amino acids increase in tomato roots under LK (Sung et al., 2015). Armengaud et al. (2009) found that the selective reduction of acidic amino acids contributes to maintaining charge balance in response to potassium-deficient conditions. Under high-N conditions, most amino acids decreased in apple leaves, and under HK conditions, most amino acids decrease in apple (Sun et al., 2021). In plants, the accumulation of free amino acids has been reported under N and P deficiency conditions (Hernández et al., 2007; Pant et al., 2015; Mo et al., 2019; Ding et al., 2021). In this study, we found that the synthesis of most amino acids was increased in apple leaves under LK stress, especially in ornithine and arginine (**Figure 5**; **Table S4**). These results show that the deficiency of macronutrients affects the accumulation of amino acids in plants.

Carbohydrate metabolism plays core roles in plant metabolism, providing energy for plant growth and development, and it acts as a bridge in the communication of proteins, lipids, and metabolism (Rolland et al., 2006). The increased content of soluble sugars, including glucose, sucrose, and fructose, in plants is a typical response to different stresses (Armengaud et al., 2004; Rosa et al., 2009; Carvalhais et al., 2011; Wang et al., 2012b; Tang et al., 2015). Zeng et al. (2018) found that most sugars are significantly upregulated under a K-deficient environment in barley roots and leaves. Under LK and HK stresses, the glucose and glycerate-3P contents also increased in apple, suggesting that increased accumulation of sugar may be one of the physiological characteristics for different K stress adaptations in plants. Sugar and potassium have a common function in regulating osmotic potential. We also identified DEGs involved in carbohydrate metabolism, especially those related to glycolysis and the TCA cycle, which were differentially expressed in response to different K stresses. Glycolysis is a process of glucose breakdown to form pyruvate (Fernie et al., 2004). Changes in the levels of gene transcripts in the glycolytic pathway, such as phosphofruckinase-1 (PFK), aldolase (AD), pyruvate-phosphate kinase (PPDK), and phosphoenolpyruvate carboxylase kinase (PPCK), were found under LK and HK conditions in apple. PFK catalyzes a reversible reaction in glycolysis and regulates the glycolysis pathway (Schaeffer et al., 1996; Mustroph et al., 2013). It has been reported that the *PFK* gene is upregulated in barley under LK (Ye et al., 2022). In this study, the expression of *PFK* (MD01G107500 and MD07G1144100) was increased in apple leaves, which may have induced an increase the glycerate-3P content in apple leaves under LK (**Figure 5**). Li et al. (2018) revealed that the TCA cycle of two soybean genotypes is inhibited in leaves and roots under low-N stress. We have previously reported that the TCA cycle is also decreased under N-deficient stress in apple leaves (Sun et al., 2021). The DEGs involved in the TCA cycle, namely, fumarase (*FUM*), isocitrate dehydrogenase (*IDH*), and citrate synthase (*CS*), were also downregulated in apple leaves under LK (**Figure 5**), indicating that LK stress caused greater damage to apple leaves. Carbohydrate metabolism enzymes, particularly those involved in glycolysis and the TCA cycle, may be indispensable for plant survival under low nutrient conditions (Zeng et al., 2015).

Combined KEGG enrichment analysis of these pathways showed that the biosynthesis of flavonoids was a significantly enriched pathway under different K stresses (**Figure 6**). Flavonoids are secondary metabolites with low molecular weights, and they are widely found in plant communities and are closely related to the UV protection, flower color formation, plant growth regulation, and pathogen resistance. Many studies have found that flavonoids are related to macronutrients in plants, such as N, P, and K. In rapeseed, nitrogen deficiency enhances *ANS* and *DFR* gene expression (Koeslin-Findeklee et al., 2015). The expression of the *PAL5*, *CHS2*, *F3’H*, and *F3’5’H* genes is significantly increased in tomato leaves under N deficiency stress (Løvdal et al., 2010). Luo et al. (2019) reported that flavonoids are significantly decreased under low P in maize. In the present study, the DEGs and DAMs involved in the flavonoid pathway also changed in apple under different K conditions (**Figure 6**). The level of naringenin chalcone in apple leaves and roots was increased under LK conditions. Moreover, the phenylalanine content decreased in apple leaves under different K conditions. The *PAL* gene in apple leaves was upregulated under LK stress but downregulated under HK conditions. The *C4H*, *4CL*, *CHS*, *F3H*, *ANS*, *CHI*, and *DFR* genes were upregulated under LK stress, but the *UGT* gene was downregulated under LK stress. Under HK conditions, the *PAL*, *UGT*, *CHI* (MD01G1118300), and *DFR* (MD11G1229100) genes were downregulated in leaves. Together, these results indicated that the flavonoid pathway plays an important role in the apple response to different potassium stresses.

## Data availability statement

The datasets presented in this study can be found in online repositories. The names of the repository/repositories and accession number(s) can be found in the article/**Supplementary Material**.

## Author contributions

BZ and QW designed the experiments. TS prepared the plant samples. TS, JKZ, and QZ conducted the experiments and analyzed the data. TS, BZ, and QW wrote the manuscript, with the help of XL, ML, YY, and JZ. All authors contributed to the article and approved the submitted version.
