# Supplementary material for: Transcriptional and metabolic responses of apple to different potassium environments
Source: Front Plant Sci. 2023 Mar 10;14:1131708. doi: 10.3389/fpls.2023.1131708 (PMC10036783; doi:10.3389/fpls.2023.1131708)
Supplement: Supplementary file 1 [file DataSheet_1.docx]

**Table S1** The primers used for qRT-PCR.

|  | **Gene** | **Forward** | **Reverse** |
| --- | --- | --- | --- |
| qRT-PCR | MD03G1121400 | 5' TAACCGCAACATTACTCCCT 3' | 5' ACATAAAAACTCCCATCCAA 3' |
|  | MD11G1052900 | 5' AGAAATGGACTCCCACAAGC 3' | 5' TGGACCCCAATTCGACCTGA 3' |
|  | MD03G1020900 | 5' ATTGCGGAGGTTAGGGAC 3' | 5' CAAACTGAGGACGAGGGG 3' |
|  | MD13G1257800 | 5' GCGGAAACTGAGGAAGACGGTA 3' | 5' AACAAGGGCAGCACGCAGAGGA 3' |
|  | MD02G1153500 | 5' GACATTATTGAACTCCCACCGC 3' | 5' AAACGTACTTAGGAATCCCCAG 3' |
|  | MD04G1003400 | 5' TGGACGAGGTGAGGAGGAAA 3' | 5' AAGCAGCCACACTGTGAAGC 3' |
|  | MD01G1118300 | 5' TCGGAGTGTACTTGGAGGAAAA 3' | 5' CAACTGAGGAAGGGCGGGCTAT 3' |
|  | MD15G1436600 | 5' TTGGTCCATTATTTCTGGTCTC 3' | 5' TCTGTAGCGTTTCTTATTTTTC 3' |
|  | MD08G1028600 | 5' CTTTGAGTCCAAGGACCCCGAG 3' | 5' AAACGAAGTACATCCAACCAGT 3' |
|  | MD11G1229100 | 5' TTCTCACTTCCAAGGTTCCATC 3' | 5' TCATTTTCTTCCTCAATCACTC 3' |
|  | EF-1α | 5' ATTCAAGTATGCCTGGGTGC 3' | 5' CAGTCAGCCTGTGATGTTCC 3' |

**Table S2** Mineral nutrients (mg/g DW) in root, stem, leaves and total concentration of apple under control (CK), low-potassium (LK) and high-potassium (HK) conditions. Data indicate means ± SE (n = 3). Different letters behind the values in the same column for each tissue indicate significant difference between the treatments.

| **Element** | **Treatment** | **The element uptake of each organ (mg/g)** | | | **Total concentration**  **(mg/g DW)** |
| --- | --- | --- | --- | --- | --- |
|  |  | **Root** | **Stem** | **Leaves** |  |
|  | CK | 11.11±0.14 b | 7.55±0.05 a | 15.29±0.33 c | 14.26±0.28 b |
| N | LK | 12.72±0.06 a | 7.62±0.03 a | 20.51±0.31 a | 14.31±0.28 b |
|  | HK | 12.65±0.14 a | 7.35±0.05 b | 19.37±0.11 b | 15.45±0.23 a |
|  |  |  |  |  |  |
|  | CK | 1.65±0.05 b | 1.53±0.02 b | 2.00±0.02 b | 1.68±0.03 b |
| P | LK | 1.86±0.04 a | 1.59±0.01 a | 2.13±0.06 a | 1.84±0.03 a |
|  | HK | 1.53±0.02 c | 1.06±0.03 c | 1.34±0.04 c | 1.61±0.03 c |
|  |  |  |  |  |  |
|  | CK | 7.30±0.16 b | 5.72±0.15 b | 11.37±0.20 b | 10.52±0.29 b |
| K | LK | 7.00±0.11 c | 4.25±0.04 c | 10.67±0.26 c | 8.63±0.11 c |
|  | HK | 7.98±0.15 a | 7.88±0.05 a | 16.48±0.12 a | 14.30±033 a |

**Table S3** Summary of the sequencing data generated for RNA-Seq and mapping on the apple genome.

| Sample | Raw Reads | Clean Reads | Mapped Reads |
| --- | --- | --- | --- |
| ck-l-1 | 50449512 | 49326390 | 44276672(89.76%) |
| ck-l-2 | 52861120 | 51692654 | 46399604(89.76%) |
| ck-l-3 | 51609594 | 50235978 | 45087916(89.75%) |
| ck-r-1 | 55152790 | 53976402 | 46461228(86.08%) |
| ck-r-2 | 56672566 | 55444912 | 47644971(85.93%) |
| ck-r-3 | 48134576 | 46895466 | 40338402(86.02%) |
| lk-l-1 | 46823658 | 46050022 | 40417041(89.59%) |
| lk-l-2 | 46468362 | 45661948 | 41406908(89.62%) |
| lk-l-3 | 44453824 | 43428748 | 41561745(89.49%) |
|  |  |  |  |
| lk-r-1 | 45983974 | 45190290 | 41340016(82.59%) |
| lk-r-2 | 51630998 | 50555930 | 36783796(83.33%) |
| lk-r-3 | 46651472 | 45485332 | 40213506(84.11%) |
| hk-l-1 | 46059604 | 45115136 | 41273188(89.63%) |
| hk-l-2 | 47540392 | 46205248 | 41116250(90.04%) |
| hk-l-3 | 47486270 | 46441502 | 38960871(89.71%) |
| hk-r-1 | 51409420 | 50051932 | 39855270(88.19%) |
| hk-r-2 | 45369316 | 44144466 | 44570044(88.16%) |
| hk-r-3 | 49113230 | 47809440 | 39976761(87.89%) |


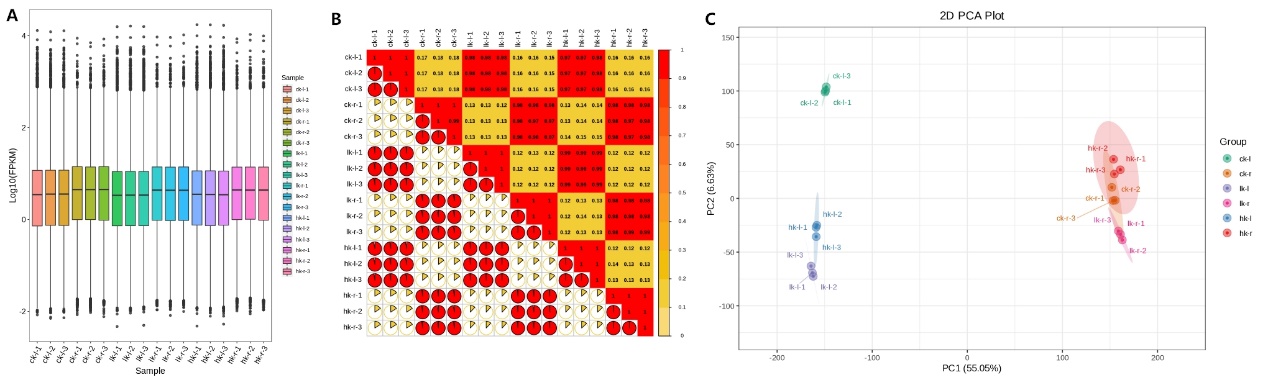


**Figure S1** (A) Distribution of the gene expression in LKL, HKL, CKL, LKR, HKR and CKR; (B) Pearson correlations in LKL/CKL, HKL/CKL, LKR/CKR and HNR/CKR; and (C) principle component analysis of expressed genes.


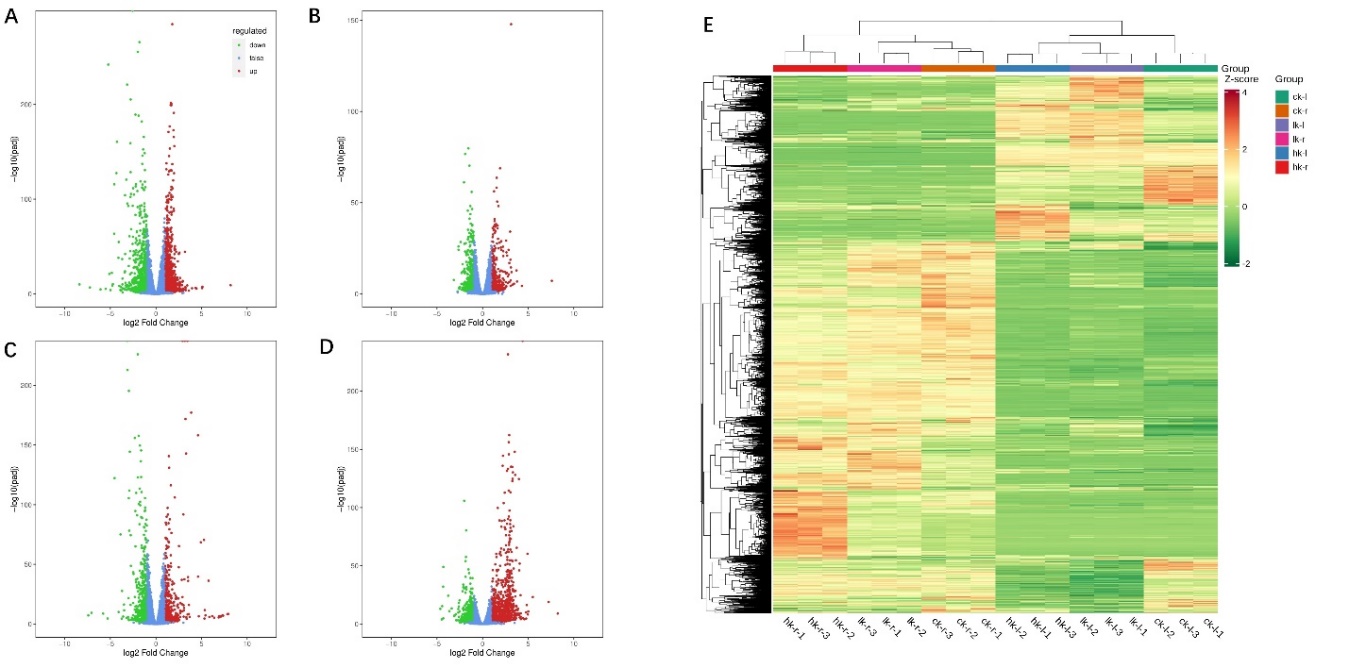


**Figure S2** (A, B, C, D) The volcano map and (E) cluster heat map of different genes in apple leaves and roots under different K conditions; the abscissa represents the sample name and hierarchical clustering results, and the ordinate represents the differential genes and hierarchical clustering results.


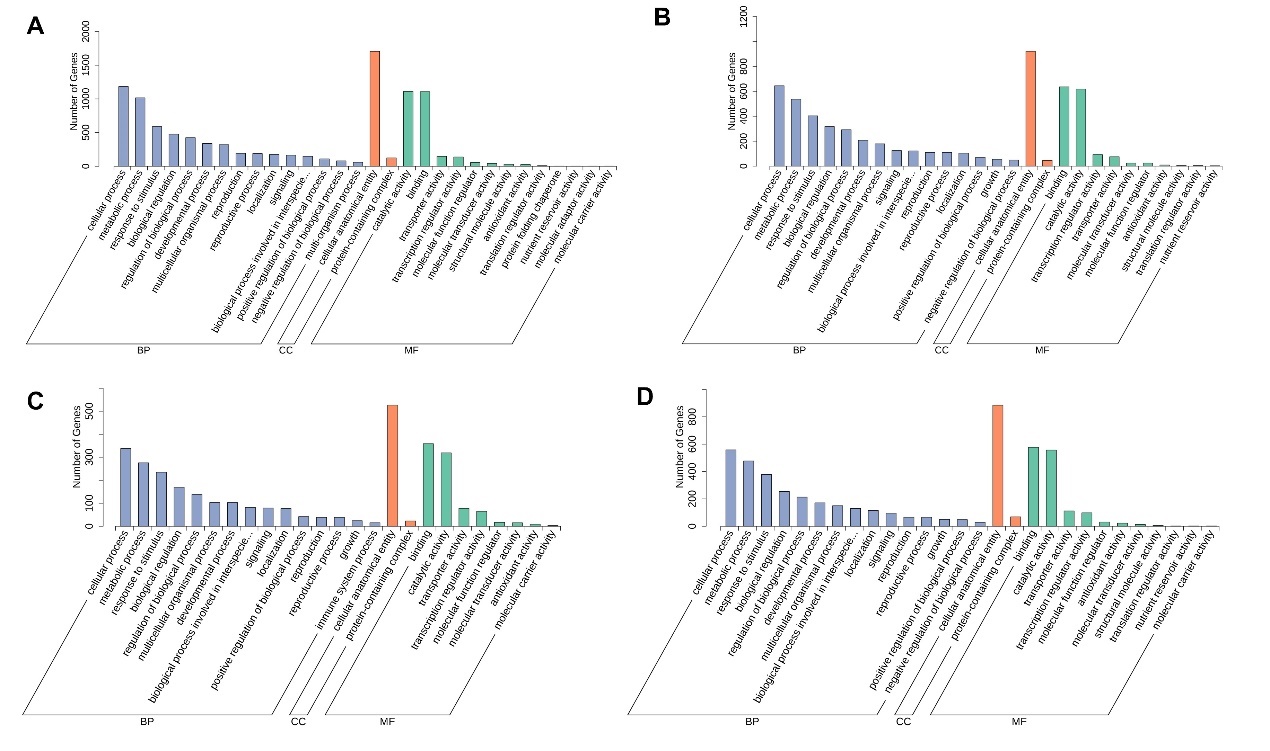


**Figure S3** GO annotation of DEGs in LKL/CKL, HKL/CKL, LKR/CKR and HKR/CKR. The x-axis indicates the GO classifications, and the Y-axis indicates the number of genes in each classification.


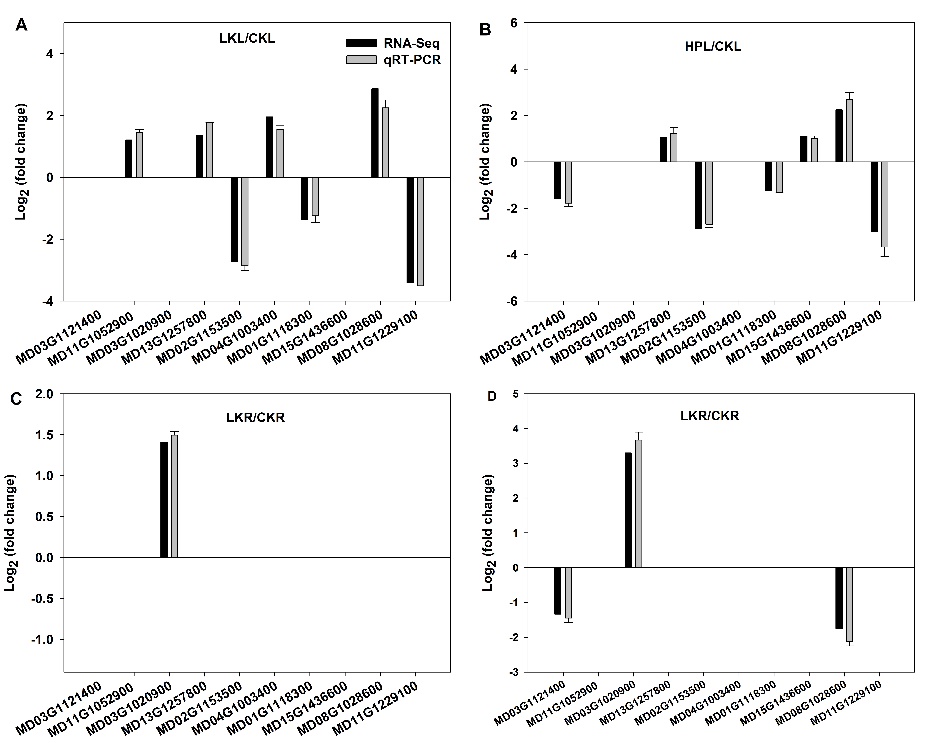


**Figure S4** qRT-PCR validation of the apple leaves and roots differentially expressed genes in different potassium treatments.
